# Di-μ-chlorido-bis­{[2-(morpholinometh­yl)phenyl-κ^2^ *C* ^1^,*N*]palladium(II)}

**DOI:** 10.1107/S1600536808022575

**Published:** 2008-07-23

**Authors:** Dana Copolovici, Cristian Silvestru, Francesco Isaia, Richard A. Varga

**Affiliations:** a Faculty of Chemistry and Chemical Engineering, Babes-Bolyai University, 11 Arany Janos Street, RO-400028, Cluj Napoca, Romania; b Universita degli Studi di Cagliari, Dipartimento di Chimica Inorganica ed Analitica, 09042 Monserrato, Cagliari, Italy

## Abstract

The title compound, [Pd_2_(C_11_H_14_NO)_2_Cl_2_], has a dimeric structure with Cl atoms bridging the two Pd atoms, one half of the mol­ecule being generated by symmetry due to the crystallographic inversion centre located in the middle of the perfectly planar Pd_2_Cl_2_ ring. The five-membered ring adopts an envelope conformation, while the morpholino group has a chair conformation. The geometry around the metal centres is distorted square-planar, as a result of a strong intra­molecular N→Pd coordination *trans* to a Pd—Cl bond. In the crystal structure, the dimeric structure is strengthened by inter­molecular C—H⋯Cl hydrogen bonds. C—H⋯C_phen­yl_ inter­actions link the dimers into a columnar supra­molecular array along the *a* axis; the dimers are further connected by C—H⋯Ph inter­actions into a three-dimensional supra­molecular arrangement.

## Related literature

For related literature, see: Copolovici *et al.* (2007 ▶, 2008 ▶); Crispini *et al.* (1992 ▶); Fuchita *et al.* (1999 ▶); Mahalakshmi *et al.* (2003 ▶); Mentes *et al.* (1997 ▶, 2004 ▶, 2005 ▶); Phadnis *et al.* (2002 ▶, 2003 ▶); Emsley (1994 ▶); IUPAC (1979 ▶).
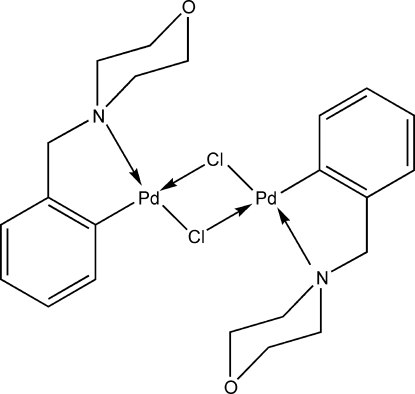


## Experimental

### 

#### Crystal data

[Pd_2_(C_11_H_14_NO)_2_Cl_2_]
*M* *_r_* = 636.20
Monoclinic, 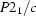

*a* = 8.1234 (6) Å
*b* = 16.4437 (13) Å
*c* = 8.8298 (7) Å
β = 101.9570 (10)°
*V* = 1153.88 (15) Å^3^
*Z* = 2
Mo *K*α radiation
μ = 1.81 mm^−1^
*T* = 297 (2) K
0.22 × 0.17 × 0.17 mm

#### Data collection

Bruker SMART APEX CCD area-detector diffractometer
Absorption correction: multi-scan (*SADABS*; Bruker, 2000 ▶) *T*_min_ = 0.677, *T*_max_ = 0.733
9118 measured reflections
2354 independent reflections
2245 reflections with *I* > 2σ(*I*)
*R*_int_ = 0.026

#### Refinement

*R*[*F* ^2^ > 2σ(*F* ^2^)] = 0.028
*wR*(*F* ^2^) = 0.059
*S* = 1.15
2354 reflections
137 parameters
H-atom parameters constrained
Δρ_max_ = 0.49 e Å^−3^
Δρ_min_ = −0.43 e Å^−3^

### 

Data collection: *SMART* (Bruker, 2000 ▶); cell refinement: *SAINT-Plus* (Bruker, 2001 ▶); data reduction: *SAINT-Plus*; program(s) used to solve structure: *SHELXS97* (Sheldrick, 2008 ▶); program(s) used to refine structure: *SHELXL97* (Sheldrick, 2008 ▶); molecular graphics: *DIAMOND* (Brandenburg & Putz, 2006 ▶); software used to prepare material for publication: *publCIF* (Westrip, 2008 ▶).

## Supplementary Material

Crystal structure: contains datablocks I, global. DOI: 10.1107/S1600536808022575/hk2489sup1.cif

Structure factors: contains datablocks I. DOI: 10.1107/S1600536808022575/hk2489Isup2.hkl

Additional supplementary materials:  crystallographic information; 3D view; checkCIF report

## Figures and Tables

**Table:** 

Cl1—Pd1	2.3232 (8)
Cl1—Pd1^i^	2.4815 (8)
N1—Pd1	2.118 (2)
C1—Pd1	1.971 (3)

**Table:** 

Cl1—Pd1—Cl1^i^	82.66 (3)
N1—Pd1—Cl1	174.34 (7)
N1—Pd1—Cl1^i^	102.76 (6)
C1—Pd1—Cl1	92.88 (9)
C1—Pd1—Cl1^i^	175.53 (9)
C1—Pd1—N1	81.69 (11)
Pd1—Cl1—Pd1^i^	97.34 (3)
C7—N1—Pd1	104.01 (17)
C8—N1—Pd1	117.35 (18)
C11—N1—Pd1	111.92 (17)

Symmetry code: (i) 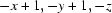.

**Table 2:** Hydrogen-bond geometry (Å, °) *Cg* is the centroid of the C1–C6 ring.

*D*—H⋯*A*	*D*—H	H⋯*A*	*D*⋯*A*	*D*—H⋯*A*
C9—H9*B*⋯Cl1^i^	0.97	2.49	3.370 (3)	150
C7—H7*B*⋯C1^ii^	0.97	2.67	3.588 (4)	159
C11—H11*A*⋯*Cg*^iii^	0.97	2.93	3.829 (3)	154

Symmetry codes: (i) 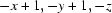; (ii) 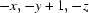; (iii) 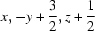.

**Fig. 1.**
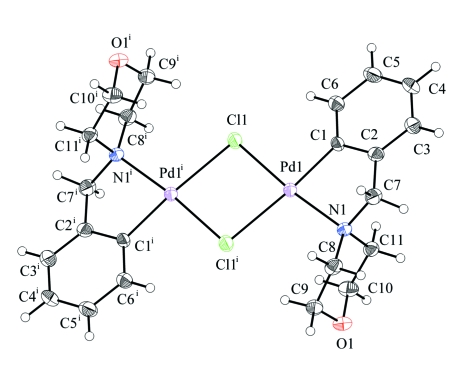
The molecular structure of the title molecule, with the atom-numbering scheme. Displacement ellipsoids are drawn at the 30% probability level [symmetry code: (i) 1 - x, 1 - y, -z].

**Fig. 2.**
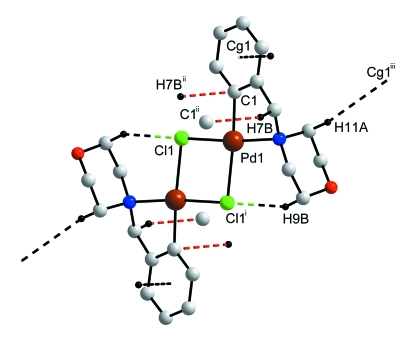
: Intermolecular interactions in the title compound. Hydrogen bonds are shown as dashed lines [symmetry codes: (i) 1 - x, 1 - y, -z; (ii) -x, 1 - y, -z; (iii) x, 3/2 - y, 1/2 + z]. H atoms not involved in hydrogen bonding are omitted for clarity.

**Fig. 3.**
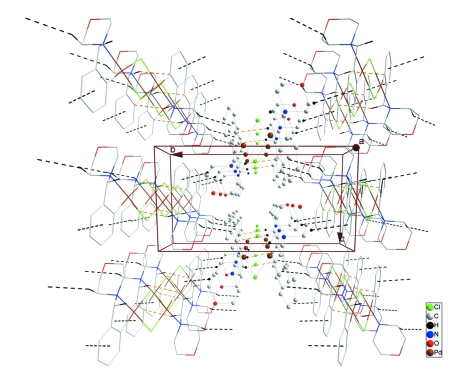
: A packing diagram of the title compound, showing the supramolecular arrangement.

## supplementary crystallographic information

### Comment

There are scarce data reported upon the reactivity of triorganoantimony(III) compounds toward palladium(II) chloride or corresponding complexes. The compounds of the type [PdCl_2_(Sb*R*_3_)_2_], *R* = *^i^*Pr (Phadnis *et al.*, 2002), *ortho*-tolyl (Mentes & Fawcett, 2005), were obtained by reaction of [PdCl_2_(COD)], COD = cycloocta-1,5-diene, with the corresponding stibines. The reaction of [PdCl_2_(PhCN)_2_] with Sb*R*_3_ (*R* = 2-thienyl) resulted in a black insoluble powder (possible Pd metal) due to a decomposition process (Mahalakshmi *et al.*, 2003), whilst the reaction of [PdCl_2_(PhCN)_2_] with tri(allyl)stibine and tris(2-methylallyl)stibine afforded the transmetalation products [Pd_2_(µ-Cl)_2_*R*_2_], *R* = allyl, 2-methylallyl, and the fate of the stibine residue is unknown (Phadnis *et al.*, 2003). Reaction of SbPh_3 _with [PdCl_2_(COD)] or PdCl_2 _afforded *trans*-[PdCl(σ-Ph)(SbPh_3_)_2_] due to the easy cleveage of Sb—C bond in SbPh_3_, whilst reaction of Na_2_PdCl_4 _with SbPh_3 _gave *cis*-[PdCl_2_(SbPh_3_)_2_] contaminated with small amount of chloro σ-phenyl complex (Mentes *et al.*, 1997). Related to our interest on the synthesis and chemical properties of organoantimony(III) compounds, we performed the reaction between Sb[C_6_H_4_CH_2_{N(CH_2_CH_2_)_2_O}-2]_3_ and [PdCl_2_(MeCN)_2_] in acetonitrile/chloroform mixture, at room temperature.

The title compound has a dimeric structure, with two palladacycles bridged through the chlorine atoms, resulting in a perfectly planar Pd_2_Cl_2_ core. One half of the molecule is generated by symmetry owing the crystallographic inversion centre located in the middle of the Pd_2_Cl_2_ ring. The cycle is distorted from the ideal square as reflected by the diferences in the Pd—Cl bond lengths and the Cl1—Pd1—Cl1^i^ and Pd1—Cl1—Pd1^i^ bond angles (Table 1) [symmetry code: (i) = 1 - *x*, 1 - *y*, -*z*].

The N atom from the pendant arm coordinates the metal centre resulting in a square planar (*C,N*)PdCl_2_ core, in which the distortion is mainly due to the PdC_3_N ring constraint. The two equivalent organic ligands from the dimer are in a *trans* arrangement with respect to the Pd···Pd axis (Fig. 1). The Pd1—C1 [1.971 (2) Å] bond is smaller than the sum of the corresponding covalent radii, its magnitude being similar to those found for related compounds for which partialy Pd—C multiple bond was assumed (Crispini *et al.*, 1992, Fuchita *et al.*, 1999, Mentes *et al.*, 2004).

An almost ideal *chair *conformation was observed for the morpholinyl groups with torsion angles [C8—N1—C11—C10 = -52.6 (3)° and C10—O1—C9—C8 = 58.4 (3)°] similar with those found in 4-benzylmorpholin-4-ium chloride (Copolovici *et al.*, 2007) and in tris[2-(morpholin-4-ylmethyl)phenyl -*κ*^2^*C*^1^,*N*]αntimony(III) (Copolovici *et al.*, 2008).

As a result of the intramolecular coordination of the N1 atom from the organic ligand to Pd1 atom, a nonplanar five-membered ring is formed, with nitrogen atom lying out of the Pd1/C1/C2/C7 best plane [0.666 (2) Å]. The dihedral angle between the Pd1/N1/C7 and Pd1/C1/C2/C7 planes is 39.2 (1)°. This induces planar chirality (with the aromatic ring and the nitrogen atom as chiral plane and pilot atom, respectively, IUPAC, 1979). In the crystal of the title compound, the dimer contains both *R*_N_ and *S*_N_^i^ isomers.

In the crystal structure, the dimer is strengthened by hydrogen-bond type interactions (Table 2) involving the Cl from one molecular unit and the methylenic proton of the morpholinyl group from the other molecular unit from the same dimer [the sum of van der Waals radii of the corresponding atoms Σr_vdW _(Cl,H) = 3.00 Å; Emsley, 1994] (Fig. 2).

Intermolecular *C*—*H*···C_phenyl_ interactions (Table 2) between a methylene hydrogen from the pendant arm and a carbon atom from the aromatic ring of another dimer connect the molecular units in a columnar arrangement along the *a* axis. Furthermore, these arrays are interlinked by intermolecular C—H···Ph interactions (Table 2) in a three-dimensional supramolecular arrangement in the crystal structure (Fig.3).

### Experimental

For the preparation of the title compound, PdCl_2_ (0.1 g, 0.56 mmol) in acetonitrile (40 ml) was refluxed for 3 h, and then allowed to cool to room temperature. A solution of Sb[C_6_H_4_CH_2_{N(CH_2_CH_2_)_2_O}-2]_3_ (0.369 g, 0.56 mmol) in CHCl_3_ (35 ml) was added and the mixture was stirred at room temperature for 30 h, under an N_2_ atmosphere. The reaction mixture was filtered off and the solvents were removed under vacuum. The yellow oil obtained was triturated with hexane (2 *x* 20 ml) and then washed with petroleum ether (2 *x* 10 ml) to give a yellow solid. Yellow crystals suitable for X-ray diffraction studies were obtained by slow diffusion of hexane into a solution of CH_2_Cl_2_ of the title compound (1:1 *v*/*v* ratio) (yield: 0.217 g, 47%). Anal. Found: C, 41.38; H, 4.63; N, 4.73. Calc. for C_22_H_28_Cl_2_N_2_O_2_Pd_2_ (636.22): C, 41.53; H, 4.44; N, 4.40%. FT—IR (KBr, cm^-1^): C—H stretch aromatic: 3038, 3035, 2958, 2891; 2857, 1436, 1114, 1069, 868, 745.

### Refinement

H atoms were positioned geometrically, with C-H = 0.93 and 0.97 Å for aromatic and methylene H, respectively, and constrained to ride on their parent atoms with U_iso_(H) = 1.2U_eq_(C),

### Crystal data

**Table:**

[Pd_2_(C_11_H_14_NO)_2_Cl_2_]	*F*_000_ = 632
*M**_r_* = 636.20	*D*_x_ = 1.831 Mg m^−^^3^
Monoclinic, *P*2_1_/*c*	Mo *K*α radiation λ = 0.71073 Å
Hall symbol: -P 2ybc	Cell parameters from 4334 reflections
*a* = 8.1234 (6) Å	θ = 2.5–27.3º
*b* = 16.4437 (13) Å	µ = 1.81 mm^−^^1^
*c* = 8.8298 (7) Å	*T* = 297 (2) K
β = 101.9570 (10)º	Block, yellow
*V* = 1153.88 (15) Å^3^	0.22 × 0.17 × 0.17 mm
*Z* = 2	

### Data collection

**Table:**

Bruker SMART APEX CCD area-detector diffractometer	2354 independent reflections
Radiation source: fine-focus sealed tube	2245 reflections with *I* > 2σ(*I*)
Monochromator: graphite	*R*_int_ = 0.026
*T* = 297(2) K	θ_max_ = 26.4º
φ and ω scans	θ_min_ = 2.5º
Absorption correction: multi-scan(SADABS; Bruker, 2000)	*h* = −10→10
*T*_min_ = 0.677, *T*_max_ = 0.733	*k* = −20→20
9118 measured reflections	*l* = −11→11

### Refinement

**Table:**

Refinement on *F*^2^	Secondary atom site location: difference Fourier map
Least-squares matrix: full	Hydrogen site location: inferred from neighbouring sites
*R*[*F*^2^ > 2σ(*F*^2^)] = 0.028	H-atom parameters constrained
*wR*(*F*^2^) = 0.059	*w* = 1/[σ^2^(*F*_o_^2^) + (0.0166*P*)^2^ + 1.0911*P*] where *P* = (*F*_o_^2^ + 2*F*_c_^2^)/3
*S* = 1.15	(Δ/σ)_max_ = 0.002
2354 reflections	Δρ_max_ = 0.49 e Å^−^^3^
137 parameters	Δρ_min_ = −0.43 e Å^−^^3^
Primary atom site location: structure-invariant direct methods	Extinction correction: none

### Special details

**Table:**

Geometry. All e.s.d.'s (except the e.s.d. in the dihedral angle between two l.s. planes) are estimated using the full covariance matrix. The cell e.s.d.'s are taken into account individually in the estimation of e.s.d.'s in distances, angles and torsion angles; correlations between e.s.d.'s in cell parameters are only used when they are defined by crystal symmetry. An approximate (isotropic) treatment of cell e.s.d.'s is used for estimating e.s.d.'s involving l.s. planes.
Refinement. Refinement of F^2^ against ALL reflections. The weighted R-factor wR and goodness of fit S are based on F^2^, conventional R-factors R are based on F, with F set to zero for negative F^2^. The threshold expression of F^2^ > 2sigma(F^2^) is used only for calculating R-factors(gt) etc. and is not relevant to the choice of reflections for refinement. R-factors based on F^2^ are statistically about twice as large as those based on F, and R- factors based on ALL data will be even larger.

### Fractional atomic coordinates and isotropic or equivalent isotropic displacement parameters (Å^2^)

**Table:**

	*x*	*y*	*z*	*U*_iso_*/*U*_eq_	
Pd1	0.32057 (3)	0.559622 (13)	0.02382 (2)	0.03252 (8)	
Cl1	0.41182 (11)	0.49501 (6)	−0.17788 (9)	0.0591 (3)	
O1	0.4523 (3)	0.69670 (15)	0.4417 (3)	0.0524 (6)	
N1	0.2135 (3)	0.61896 (14)	0.1932 (3)	0.0311 (5)	
C1	0.1119 (3)	0.59843 (17)	−0.1126 (3)	0.0332 (6)	
C2	−0.0122 (4)	0.62200 (19)	−0.0349 (4)	0.0382 (7)	
C3	−0.1640 (4)	0.6534 (2)	−0.1153 (4)	0.0564 (10)	
H3	−0.2460	0.6700	−0.0624	0.068*	
C4	−0.1921 (4)	0.6598 (2)	−0.2752 (4)	0.0578 (10)	
H4	−0.2932	0.6811	−0.3296	0.069*	
C5	−0.0721 (4)	0.6349 (2)	−0.3534 (4)	0.0494 (8)	
H5	−0.0930	0.6382	−0.4608	0.059*	
C6	0.0798 (4)	0.60484 (19)	−0.2732 (4)	0.0401 (7)	
H6	0.1614	0.5888	−0.3270	0.048*	
C7	0.0286 (4)	0.6089 (2)	0.1360 (4)	0.0426 (7)	
H7A	−0.0318	0.6480	0.1864	0.051*	
H7B	−0.0055	0.5547	0.1601	0.051*	
C8	0.2571 (4)	0.5867 (2)	0.3546 (3)	0.0437 (8)	
H8A	0.2506	0.5278	0.3510	0.052*	
H8B	0.1743	0.6059	0.4112	0.052*	
C9	0.4285 (4)	0.6111 (2)	0.4406 (4)	0.0493 (8)	
H9A	0.4448	0.5917	0.5464	0.059*	
H9B	0.5125	0.5854	0.3929	0.059*	
C10	0.4272 (4)	0.7249 (2)	0.2865 (4)	0.0497 (8)	
H10A	0.5065	0.6983	0.2344	0.060*	
H10B	0.4479	0.7830	0.2862	0.060*	
C11	0.2517 (4)	0.70759 (18)	0.2014 (3)	0.0378 (7)	
H11A	0.1730	0.7351	0.2529	0.045*	
H11B	0.2365	0.7292	0.0972	0.045*	

### Atomic displacement parameters (Å^2^)

**Table:**

	*U*^11^	*U*^22^	*U*^33^	*U*^12^	*U*^13^	*U*^23^
Pd1	0.03461 (14)	0.03664 (14)	0.02611 (12)	0.00850 (9)	0.00582 (9)	0.00045 (9)
Cl1	0.0574 (5)	0.0891 (7)	0.0284 (4)	0.0394 (5)	0.0034 (4)	−0.0061 (4)
O1	0.0565 (14)	0.0596 (15)	0.0356 (12)	0.0015 (12)	−0.0028 (10)	−0.0127 (11)
N1	0.0336 (12)	0.0330 (12)	0.0270 (12)	−0.0006 (10)	0.0071 (10)	−0.0009 (10)
C1	0.0313 (14)	0.0282 (14)	0.0368 (16)	0.0048 (12)	−0.0006 (12)	0.0000 (12)
C2	0.0324 (15)	0.0417 (17)	0.0391 (17)	−0.0050 (13)	0.0039 (13)	−0.0066 (13)
C3	0.0319 (17)	0.077 (3)	0.057 (2)	0.0086 (17)	0.0022 (16)	−0.0190 (19)
C4	0.0371 (18)	0.069 (2)	0.057 (2)	0.0090 (17)	−0.0122 (16)	−0.0084 (19)
C5	0.0468 (19)	0.056 (2)	0.0398 (18)	0.0014 (16)	−0.0038 (15)	0.0025 (16)
C6	0.0390 (16)	0.0432 (17)	0.0365 (16)	0.0032 (14)	0.0043 (13)	0.0025 (14)
C7	0.0344 (16)	0.0509 (19)	0.0446 (18)	−0.0057 (14)	0.0131 (14)	−0.0087 (15)
C8	0.060 (2)	0.0444 (18)	0.0291 (16)	0.0036 (16)	0.0144 (15)	0.0038 (13)
C9	0.059 (2)	0.059 (2)	0.0274 (16)	0.0164 (17)	0.0032 (15)	0.0011 (15)
C10	0.050 (2)	0.050 (2)	0.0455 (19)	−0.0111 (16)	0.0020 (15)	−0.0035 (16)
C11	0.0464 (17)	0.0309 (15)	0.0343 (16)	0.0022 (13)	0.0042 (13)	−0.0015 (12)

### Geometric parameters (Å, °)

**Table:**

Pd1—Cl1^i^	2.4815 (8)	C7—H7A	0.9700
Cl1—Pd1	2.3232 (8)	C7—H7B	0.9700
Cl1—Pd1^i^	2.4815 (8)	C8—N1	1.493 (4)
N1—Pd1	2.118 (2)	C8—C9	1.496 (5)
C1—C2	1.387 (4)	C8—H8A	0.9700
C1—C6	1.392 (4)	C8—H8B	0.9700
C1—Pd1	1.971 (3)	C9—O1	1.421 (4)
C2—C3	1.389 (4)	C9—H9A	0.9700
C2—C7	1.492 (4)	C9—H9B	0.9700
C3—C4	1.387 (5)	C10—O1	1.421 (4)
C3—H3	0.9300	C10—C11	1.495 (4)
C4—C5	1.369 (5)	C10—H10A	0.9700
C4—H4	0.9300	C10—H10B	0.9700
C5—C6	1.381 (4)	C11—N1	1.489 (4)
C5—H5	0.9300	C11—H11A	0.9700
C6—H6	0.9300	C11—H11B	0.9700
C7—N1	1.492 (4)		
			
Cl1—Pd1—Cl1^i^	82.66 (3)	C1—C6—H6	119.7
N1—Pd1—Cl1	174.34 (7)	N1—C7—C2	108.9 (2)
N1—Pd1—Cl1^i^	102.76 (6)	N1—C7—H7A	109.9
C1—Pd1—Cl1	92.88 (9)	C2—C7—H7A	109.9
C1—Pd1—Cl1^i^	175.53 (9)	N1—C7—H7B	109.9
C1—Pd1—N1	81.69 (11)	C2—C7—H7B	109.9
Pd1—Cl1—Pd1^i^	97.34 (3)	H7A—C7—H7B	108.3
C10—O1—C9	108.9 (2)	N1—C8—C9	113.6 (3)
C7—N1—C8	107.9 (2)	N1—C8—H8A	108.8
C7—N1—Pd1	104.01 (17)	C9—C8—H8A	108.8
C8—N1—Pd1	117.35 (18)	N1—C8—H8B	108.8
C11—N1—Pd1	111.92 (17)	C9—C8—H8B	108.8
C11—N1—C7	108.0 (2)	H8A—C8—H8B	107.7
C11—N1—C8	107.2 (2)	O1—C9—C8	112.4 (3)
C2—C1—C6	118.8 (3)	O1—C9—H9A	109.1
C2—C1—Pd1	114.2 (2)	C8—C9—H9A	109.1
C6—C1—Pd1	127.1 (2)	O1—C9—H9B	109.1
C1—C2—C3	120.7 (3)	C8—C9—H9B	109.1
C1—C2—C7	115.4 (3)	H9A—C9—H9B	107.9
C3—C2—C7	123.9 (3)	O1—C10—C11	110.8 (3)
C4—C3—C2	119.4 (3)	O1—C10—H10A	109.5
C4—C3—H3	120.3	C11—C10—H10A	109.5
C2—C3—H3	120.3	O1—C10—H10B	109.5
C5—C4—C3	120.4 (3)	C11—C10—H10B	109.5
C5—C4—H4	119.8	H10A—C10—H10B	108.1
C3—C4—H4	119.8	N1—C11—C10	112.2 (3)
C4—C5—C6	120.2 (3)	N1—C11—H11A	109.2
C4—C5—H5	119.9	C10—C11—H11A	109.2
C6—C5—H5	119.9	N1—C11—H11B	109.2
C5—C6—C1	120.5 (3)	C10—C11—H11B	109.2
C5—C6—H6	119.7	H11A—C11—H11B	107.9
			
Pd1^i^—Cl1—Pd1—C1	−179.66 (9)	C1—C2—C7—N1	32.1 (4)
Pd1^i^—Cl1—Pd1—Cl1^i^	0.0	C3—C2—C7—N1	−149.6 (3)
C11—N1—Pd1—C1	−84.42 (19)	N1—C8—C9—O1	54.4 (4)
C7—N1—Pd1—C1	31.98 (19)	O1—C10—C11—N1	−61.0 (4)
C8—N1—Pd1—C1	151.0 (2)	C10—C11—N1—C7	168.6 (3)
C11—N1—Pd1—Cl1^i^	96.05 (18)	C10—C11—N1—C8	52.6 (3)
C7—N1—Pd1—Cl1^i^	−147.55 (17)	C10—C11—N1—Pd1	−77.4 (3)
C8—N1—Pd1—Cl1^i^	−28.5 (2)	C2—C7—N1—C11	77.7 (3)
C6—C1—C2—C3	−1.6 (5)	C2—C7—N1—C8	−166.7 (3)
Pd1—C1—C2—C3	177.7 (3)	C2—C7—N1—Pd1	−41.4 (3)
C6—C1—C2—C7	176.8 (3)	C9—C8—N1—C11	−49.5 (3)
Pd1—C1—C2—C7	−3.9 (3)	C9—C8—N1—C7	−165.6 (3)
C1—C2—C3—C4	1.1 (5)	C9—C8—N1—Pd1	77.4 (3)
C7—C2—C3—C4	−177.1 (3)	C11—C10—O1—C9	61.6 (4)
C2—C3—C4—C5	0.4 (6)	C8—C9—O1—C10	−58.5 (4)
C3—C4—C5—C6	−1.4 (6)	C2—C1—Pd1—N1	−16.4 (2)
C4—C5—C6—C1	0.9 (5)	C6—C1—Pd1—N1	162.9 (3)
C2—C1—C6—C5	0.6 (5)	C2—C1—Pd1—Cl1	162.0 (2)
Pd1—C1—C6—C5	−178.6 (2)	C6—C1—Pd1—Cl1	−18.7 (3)

Symmetry codes: (i) −*x*+1, −*y*+1, −*z*.

### Hydrogen-bond geometry (Å, °)

**Table:**

*D*—H···*A*	*D*—H	H···*A*	*D*···*A*	*D*—H···*A*
C9—H9B···Cl1^i^	0.97	2.49	3.370 (3)	150
C7—H7B···C1^ii^	0.97	2.67	3.588 (4)	159
C11—H11A···Cg^iii^	0.97	2.93	3.829 (3)	154

Symmetry codes: (i) −*x*+1, −*y*+1, −*z*; (ii) −*x*, −*y*+1, −*z*; (iii) *x*, −*y*+3/2, *z*+1/2.
